# Effects of Regular Aerobic Exercise and Resistance Training on High-Density Lipoprotein Cholesterol Levels in Taiwanese Adults

**DOI:** 10.3390/ijerph16112003

**Published:** 2019-06-05

**Authors:** Chun-Sheng Hsu, Shin-Tsu Chang, Oswald Ndi Nfor, Kuan-Jung Lee, Shiuan-Shinn Lee, Yung-Po Liaw

**Affiliations:** 1 Department of Physical Medicine and Rehabilitation, Taichung Veterans General Hospital, Taichung City 40705, Taiwan; ndmc_123r@yahoo.com.tw (C.-S.H.); ccdivlaser1959@gmail.com (S.-T.C.); 2 Department of Public Health and Institute of Public Health, Chung Shan Medical University, Taichung City 40201, Taiwan; nforoswald2@yahoo.com (O.N.N.); jasminemachi@gmail.com (K.-J.L.); 3 Department of Rehabilitation Science, Jenteh Junior College of Medicine, Nursing and Management, Miaoli County 35664, Taiwan; 4 School of Medicine, Chung Shan Medical University, Taichung City 40201, Taiwan; 5 Department of Physical Medicine and Rehabilitation, Tri-Service General Hospital, School of Medicine, National Defense Medical Center, Taipei City 11490, Taiwan; 6 Department of Family and Community Medicine, Chung Shan Medical University Hospital, Taichung City 40201, Taiwan

**Keywords:** HDL, aerobic exercise, resistance training, Taiwan

## Abstract

Increased levels of high-density lipoprotein cholesterol (HDL-C) can improve endothelial function. This may help reduce cardiovascular risks and mortality. Evidence has been provided on the association between cardiometabolic traits, such as HDL-C and exercise modalities. However, there is the absence of studies investigating this association in Taiwan. We assessed the relationship between exercise type and HDL-C among Taiwanese adults. Data were collected from Taiwan Biobank (TWB), a national biomedical research database that contains the genetic information of ethnic Taiwanese residents gathered from 2008 to 2016. We enrolled 24,856 participants aged 30 to 70 years who completed a questionnaire about their recent health behaviors including smoking, drinking, and exercise. Regular exercise was categorized as non-aerobic exercise (separated as weight training, ball game, and mixed exercise) and strict aerobic exercise. Linear regression models were used to assess the effects of exercise in a questionnaire-based manner. After multivariate adjustments, HDL-C was positively associated with aerobic (β = 1.33748, *p* < 0.0001) and non-aerobic (β = 2.56210; *p* < 0.0001) exercise. Positive associations were also found for resistance training (β = 4.01828, *p* = 0.0020), ballgame (β = 2.43815, *p* = 0.0001), and mixed exercise (β = 2.47021, *p* < 0.0001). This study demonstrated that both aerobic and non-aerobic exercise have positive effects on HDL-C among Taiwanese adults. Among the non-aerobic exercise groups, resistance training had the greatest effect.

## 1. Introduction 

Physical activity has been shown to improve human health regardless of sex, ethnicity or age [1]. It is necessary to prevent and control the increasing problem of cardiovascular (CVD) and cerebrovascular diseases, which are the leading causes of death worldwide [2]. Hyperlipidemia is one of the major risk factors that have been associated with these diseases. HDL-C (high-density lipoprotein cholesterol) is one of the cardiometabolic traits that has been consistently investigated [3]. It is athero-protective [4] because of its role in reverse cholesterol transport and can improve endothelial function by promoting repair and angiogenesis [5]. It also possesses antioxidant, antithrombotic and anti-inflammatory properties. A 0.03 mmol/L increase in HDL-C has been associated with a 2 to 3% reduction in the risk of coronary heart disease [6]. 

Associations have been reported between exercise and HDL-C [7,8]. However, a few publications have found that exercise, aerobic or resistance, do not cause clinically significant changes in HDL-C [8,9,10]. Findings from another study indicated that regular exercise can improve quality of life, musculoskeletal condition, and psychological health as well as improving cardiovascular risks and mortality [11]. High levels of physical exercise were associated with higher HDL-C and a 6% decrease in the prevalence of coronary heart disease [12]. The recommended level of exercise is 150 min/week for adults. However, more days of exercise provide additional health benefits [12,13]. An extended duration of aerobic exercise (i.e., 10 min per session) can increase HDL-C level by about 1.4 mg/dL (0.036 mmol/L) [14]. As stated above, several studies with cross-sectional and longitudinal analysis have provided evidence of the association of physical activity and exercise with cardiometabolic outcomes [14,15,16]. However, there is the absence of studies investigating this relationship in Taiwan. 

As stated earlier, exercise has a positive effect on health. According to a review by the European Society of Cardiology, the actual amount of exercise required to exert a beneficial effect on blood lipid and CVD risk is still to be determined [16]. Previous works conducted in Taiwan have not focused on exercise types and lipid fractions especially HDL-C levels. The aim of this study was to investigate the relationship between HDL-C and exercise modalities (categorized as aerobic and non-aerobic exercise) in Taiwanese adults aged 30 to 70 years.

## 2. Materials and Methods 

### 2.1. Database

Data were collected from Taiwan Biobank (TWB), a national biomedical research database that contains genetic information of the general population from 2008–2016. All methods were carried out according to relevant guidelines and regulations. Data were collected using self-reported questionnaires (exercise habit, lifestyle factors, and disease history), and from blood and urine tests. Written informed consent was obtained from each participant before data were collected. The Institutional Review Board of Chung Shan Medical University approved this study.

### 2.2. Study Participants

This study enrolled 24,856 participants aged 30–70 years. General characteristics of study participants included sex, age, triglycerides (TG), low-density lipoprotein (LDL-C), high-density lipoprotein (HDL-C), systolic blood pressure (SBP), diastolic blood pressure (DBP), body mass index (BMI), waist-hip ratio, and body fat. Lifestyle factors included alcohol consumption, exercise, and smoking. 

Information on exercise (type, frequency and time) was self-reported. Exercise types included jogging, strolling, rope jumping, swimming, gymnastics, yoga, Taijiquan, Gigong, Chinese martial arts, hiking, biking, badminton, table tennis, soccer, golf, tennis, basketball, other ball games, weight training, aerobic dance, ballroom dance, and hula hooping. Participates selected a maximum of three habitual exercise types in the questionnaires. Regular exercisers included participants who had at least 3 times of exercise per week lasting at least 30 min each time. Physical activity was classified as no exercise, aerobic and non-aerobic exercise. Furthermore, non-aerobic exercise included ball game, weight training, and mixed exercise. Examples of mixed exercise included ball game and weight training, weight training and aerobic exercise, ball game, and aerobic exercise, or all of the above. 

### 2.3. Statistical Analysis

Data management and statistical analyses were performed using the SAS 9.4 software (SAS Institute, Cary, NC, USA). All participants were divided into five exercise categories as follows: no exercise, aerobic exercise, ball game, resistance training, and mixed exercise, respectively. One-way ANOVA was used to compare HDL-C between different exercise groups. Multivariate linear regression models were used to determine the β coefficients and their *p*-values. Data were presented as means ± standard error (continuous variables). 

## 3. Results

Overall, 24,856 participants were enrolled in this study (Table 1). Exercise types included strict aerobic exercise (n = 7545), ball game (n = 327), resistance training (n = 69), and mixed exercise (n = 670). There were significant differences in HDL-C concentrations of individuals in various exercise groups (*p* < 0.0001). The mean HDL-C concentration (SE) was 54.61 mg/dL (0.16) for aerobic exercise, 53.28 mg/dL (0.70) for ball game, 54.83 mg/dL (1.53) for resistance training, 53.32 mg/dL (0.51) for mixed exercise, and 52.58 mg/dL (0.10 mg/dL) for no exercise. In general, female participants had a higher concentration of HDL-C compared to their male counterparts. 

After multivariate adjustments, HDL-C was positively associated with both aerobic (β = 1.33748, *p* < 0.0001) and non-aerobic (β = 2.56210; *p* < 0.0001) exercise (Table 2). Positive associations were also found for resistance training (β = 4.01828, *p* = 0.0020), ballgame (β = 2.43815, *p* < 0.0001), and mixed exercise (β = 2.47021, *p* < 0.0001) as shown in Table 3. The strongest association was found between HDL-C and weight training compared to all other exercise groups. Negative associations were found for current smoking (β= −2.06691, *p* < 0.0001) and BMI (i.e., β = −3.41565 for overweight and −4.72494 for obesity). Current drinking was positively associated with HDL-C (β= 5.15666, *p* < 0.0001).

## 4. Discussion

To our knowledge, this is one of the large studies to investigate the impact of aerobic and non-aerobic exercise on HDL-C in Taiwanese populations. We found that HDL-C was positively associated with aerobic and non-aerobic exercise. However, non-aerobic exercise showed a stronger association than aerobic exercise (β = 2.56210, *p* < 0.0001 vs. 1.33748, *p* < 0.0001). When analyzed by different types of non-aerobic exercise, resistance training showed the strongest association with HDL-C (β = 4.01828, *p* = 0.0020), followed by mixed exercise (β = 2.47021, *p* < 0.0001). 

In a previous study, a 13% rise in HDL-C concentration was noted after 10 weeks aerobic exercise of at least three times a week [17]. In another study, Leon and colleagues found that HDL-C could be increased by 4.6% after 12 weeks or more of aerobic exercise [18]. Moderate-intensity physical exercise of at least 150 min a week improves blood lipid profile [10]. However, a single session of aerobic exercise for 30 min or longer was found to attenuate postprandial metabolism within 12–36 hours [19]. 

Lower levels of HDL-C contribute to the development of CVD. Previous studies have suggested that higher levels of physical exercise would help to increase HDL-C, which in turn reduces the prevalence rate of CVD [11,12,20,21]. Aerobic exercise can improve cardiovascular function and endurance. The higher the frequency of exercise, the greater the health benefits. Regular aerobic exercise (without changes in dietary habits) can increase HDL-C levels by about 4.3% in most individuals [18]. An elevation of 2.53 mg/dL of HDL-C (independent of body weight), was observed after 3–5 times of weekly aerobic exercise lasting 40 min per session and for at least 27 weeks [22]. 

Mechanisms through which aerobic exercise can induce cardioprotection are yet to be clearly established. Exercise-induced HDL-C may be associated with increased apolipoprotein A1 (APOA1) production, reduced hepatic lipase activity, and decreased HDL-C catabolism. APOA1 is the main protein component that synthesizes HDL-C [22,23]. It also initiates the reverse cholesterol pathway and modulates glucose turnover.

Only a few of the studies analyzing the relationship between aerobic exercise and HDL-C have discussed the beneficial effect of resistance training in general populations. Based on a previous study, resistance training reduced LDL-C, TG, and non-HDL-C, but had no significant effect on HDL-C [12]. On the contrary, other studies have concluded that resistance training is beneficial in increasing HDL-C levels [22,24,25,26]. Tomeleri and his team found that HDL-C increased by 13.2% after eight weeks of resistance training of at least three times per week [24]. 

In the current study, we found that people who were engaged in regular resistance training had higher levels of HDL-C than those who never exercised. Surprisingly, participants that were engaged in the regular aerobic exercise had lower HDL-C compared to those that were engaged in regular weight training. So far, similar findings have not been previously reported. Resistance exercise not only reduced diseases such as CVD, metabolic syndrome, depression, osteoporosis, and sarcopenia; but also modified lipid profile including HDL-C [22,25,26]. Unlike aerobic exercise, resistance training is beneficial in that, improves muscle strength and increases bone mineral density [27]. The APOB/APOA1 ratio, an effective predictor of coronary heart disease risk significantly decreased by about 8% after resistance training [26]. Resistance exercise can also improve motor unit activation and increase secretion of lipolytic hormones [28].

Based on our data, non-aerobic exercise—special resistance training—appeared to show a stronger association with HDL-C than aerobic exercise. However, resistance training might have included mainly individuals who were engaged in home gym exercise and who might have had higher total energy expenditures, higher exercise volume or intensity. In addition, HDL-C concentration in participants who had mixed exercise was higher compared to the “no exercise and aerobic exercise groups.” These findings demonstrated that aerobic exercise together with resistance training might significantly improve HDL-C concentrations. 

We found no significant differences in HDL-C concentrations of individuals in the no exercise and ball game groups. These results suggest that HDL-C levels might not be sufficiently increased by engaging mainly in ball games, which serve mainly as a leisure-time activity for adults. 

To our knowledge, this study is the first to compare the effects of aerobic exercise and resistance training on serum HDL-C concentration in Taiwan. The influence on HDL-C was based on long-term exposure of exercise and adjustments were made for several variables. However, the study design is cross-sectional, hence, causal conclusions could not be drawn. Second, exercise intensity is emerging as the most potent prescriptive factor in disease modification. However, our database did not have the appropriate intensity and energy expenditure measures. Finally, dietary intake was not included in our analysis. Moreover, this is an observational study, hence there is little mechanistic understanding of the exposure-outcome relationship.

## 5. Conclusions

In summary, we demonstrated that self-reported regular aerobic exercise and non-aerobic exercise particularly resistance training have positive effects on HDL-C among adults in Taiwan. These activities could be encouraged for they may help to prevent the risk of CVD. 

## Figures and Tables

**Table 1:** Basic descriptive characteristics of participants according to exercise types.

Variable	No Exercise (n = 16,245)	Aerobic Exercise (n = 7545)	Non-Aerobic			*p*-Value
			Ball Game (n = 327)	Resistance Training (n = 69)	Mixed (n = 670)	
**HDL-C**	**52.58 ± 0.10**	**54.61 ± 0.16**	53.28 ± 0.70	54.83 ± 1.53	53.32 ± 0.51	<0.0001
Sex						
Female	57.18 ± 0.13	58.86 ± 0.21	61.38 ± 1.55	60.39 ± 2.80	61.80 ± 1.12	<0.0001
Male	46.84 ± 0.12	49.26 ± 0.20	50.70 ± 0.71	52.04 ± 1.69	50.96 ± 0.53	<0.0001
**WHR**						
male <0.9; female < 0.8	54.36 ± 0.16	56.41 ± 0.27	54.42 ± 1.00	54.03 ± 1.74	54.23 ± 0.66	<0.0001
male ≥ 0.9; female ≥ 0.8	51.43 ± 0.13	53.52 ± 0.19	51.98 ± 0.97	55.70 ± 2.60	51.86 ± 0.81	<0.0001
**Body Fat Rate**						
Normal	54.77 ± 0.15	56.19 ± 0.22	53.87 ± 0.88	58.26 ± 1.97	54.27 ± 0.61	<0.0001
Overweight	50.27 ± 0.13	52.65 ± 0.21	52.14 ± 1.16	50.37 ± 2.18	51.04 ± 0.94	<0.0001
**Age**						
30–40	52.93 ± 0.16	54.97 ± 0.40	52.34 ± 1.23	56.33 ± 2.01	53.27 ± 0.80	<0.0001
41–50	52.31 ± 0.19	55.04 ± 0.33	55.01 ± 1.64	56.70 ± 4.15	52.40 ± 1.01	<0.0001
51–60	52.65 ± 0.22	54.95 ± 0.26	52.73 ± 1.29	52.79 ± 2.70	54.11 ± 1.16	<0.0001
61–70	51.97 ± 0.32	53.56 ± 0.30	53.18 ± 1.49	37.00 ± 1.00	53.69 ± 1.32	0.0014
**BMI**						
Underweight	63.81 ± 0.56	67.32 ± 0.99	56.67 ± 4.89	73.00	65.75 ± 5.13	0.0227
Normal	56.80 ± 0.14	58.47 ± 0.22	56.50 ± 1.11	58.91 ± 2.11	58.44 ± 0.77	<0.0001
Overweight	49.30 ± 0.17	51.24 ± 0.25	50.89 ± 1.13	52.30 ± 1.95	50.06 ± 0.78	<0.0001
Obese	45.68 ± 0.17	47.29 ± 0.29	48.88 ± 1.21	46.92 ± 4.43	45.93 ± 0.77	<0.0001
**Smoking**						
Never	54.22 ± 0.12	56.12 ± 0.18	54.74 ± 0.86	58.12 ± 2.02	54.79 ± 0.63	<0.0001
Former	48.50 ± 0.28	49.94 ± 0.37	50.73 ± 1.60	53.58 ± 2.54	50.49 ± 1.04	0.0060
Current	46.48 ± 0.24	47.66 ± 0.51	48.73 ± 1.58	45.79 ± 2.50	49.88 ± 1.41	0.0223
**Drinking**						
Never	52.82 ± 0.11	54.93 ± 0.16	53.37 ± 0.77	55.34 ± 1.56	52.94 ± 0.54	<0.0001
Former	46.71 ± 0.58	47.31 ± 0.74	51.60 ± 3.21	40.50 ± 2.50	49.83 ± 0.54	0.4381
Current	51.41 ± 0.40	54.01 ± 0.61	53.00 ± 2.10	52.50 ± 14.50	57.43 ± 1.89	0.0002
**LDL-C**						
<130	53.15 ± 0.13	54.79 ± 0.21	53.66 ± 0.96	55.45 ± 2.08	54.03 ± 0.67	<0.0001
≥130	51.58 ± 0.15	54.29 ± 0.23	52.65 ± 0.97	53.97 ± 2.26	52.03 ± 0.76	<0.0001
**Triglyceride**						
<150	55.30 ± 0.11	57.17 ± 0.17	55.35 ± 0.77	56.89 ± 1.66	55.69 ± 0.55	<0.0001
≥150	42.70 ± 0.15	44.20 ± 0.23	44.77 ± 1.19	45.00 ± 2.34	42.35 ± 0.82	<0.0001
**SBP**						
<120	54.41 ± 0.13	56.54 ± 0.22	54.63 ± 0.97	58.02 ± 1.76	55.11 ± 0.73	<0.0001
120–139	49.68 ± 0.18	52.44 ± 0.26	51.23 ± 1.23	49.18 ± 3.22	51.42 ± 0.80	<0.0001
≥140	49.25 ± 0.30	52.47 ± 0.41	52.50 ± 1.74	48.00 ± 4.02	52.40 ± 1.51	<0.0001
**DBP**						
<80	54.06 ± 0.12	55.77 ± 0.19	54.52 ± 0.86	56.62 ± 1.73	54.71 ± 0.60	<0.0001
80–89	48.72 ± 0.21	52.34 ± 0.33	50.01 ± 1.31	52.65 ± 3.51	50.81 ± 1.11	<0.0001
≥90	47.53 ± 0.33	50.23 ± 0.54	51.48 ± 2.44	45.40 ± 4.86	48.87 ± 1.81	0.0002

HDL-C, high-density lipoprotein cholesterol is presented as mean ± SE; bold: values shown in the table represent the mean HDL-C levels of individuals in the different exercise groups. Other variables are presented as numbers (%). BMI, body mass index (measured in kg/m^2^), WHR, waist–hip ratio, total cholesterol (mg/dL), TG, triglyceride (mg/dL); LDL-C, low density lipoprotein cholesterol (mg/dL); SBP, systolic blood pressure (mmHg); DBP, diastolic blood pressure (mmHg).

**Table 2:** Multiple linear regression analysis of factors associated with HDL-C concentration including aerobic and non-aerobic exercise.

Variable	β-Coefficient	SE	*p*-Value
**Exercise (Ref: no exercise)**			
Aerobic	1.33748	0.15826	<0.0001
Non-aerobic	2.56210	0.34496	<0.0001
**Sex (Ref: female)**			
male	−8.73486	0.18737	<0.0001
**WHR (Ref: male < 0.9; female < 0.8)**			
Male > 0.9; female ≥ 0.8	−2.43206	0.16432	<0.0001
**Body fat rate (Ref: Normal)**			
Overweight	−1.76628	0.18631	<0.0001
**Age (Ref: 30–40)**			
41–50	−0.76063	0.18391	<0.0001
51–60	1.44934	0.19494	<0.0001
61–70	0.93248	0.24226	0.0001
**BMI (Ref: Normal)**			
Underweight	4.86672	0.40332	<0.0001
Overweight	−3.41607	0.18466	<0.0001
Obese	−4.72790	0.23980	<0.0001
**Smoking (Ref: Never)**			
Former	−0.16073	0.23368	0.4916
Current	−2.06155	0.23941	<0.0001
**Drinking (Ref: Never)**			
Former	−0.41432	0.43680	0.3429
Current	5.15102	0.28672	<0.0001
**LDL-C (Ref: <130)**			
≥130	1.06658	0.14539	<0.0001
**Triglyceride (Ref: <150)**			
≥150	−8.52164	0.18090	<0.0001
**SBP (Ref: <120)**			
120–139	−0.19182	0.18188	0.2916
≥140	0.32595	0.30045	0.2780
**DBP (Ref: <80)**			
80–89	0.05259	0.20426	0.7968
≥90	0.25113	0.33033	0.4471

HDL-C: high-density lipoprotein cholesterol.

**Table 3:** Multiple linear regression analysis of factors associated with HDL-C concentration including aerobic exercise, ball game, resistance training, and mixed exercise.

Variable	β-Coefficient	SE	*p*-Value
**Exercise (Ref: no exercise)**			
Aerobic	1.33557	0.15827	<0.0001
Ball game	2.43815	0.60416	<0.0001
Resistance training	4.01828	1.30055	0.0020
Mixed	2.47021	0.42890	<0.0001
**Sex (Ref: female)**			
male	−8.73348	0.18738	<0.0001
**WHR (Ref: male < 0.9; female < 0.8)**			
male > 0.9; female ≥ 0.8	−2.43365	0.16434	<0.0001
**Body fat rate (Ref: Normal)**			
Overweight	−1.76888	0.18633	<0.0001
**Age (Ref:30–40)**			
41–50	0.76651	0.18400	<0.0001
51–60	1.45484	0.19505	<0.0001
61–70	0.94048	0.24244	0.0001
**BMI (Ref: Normal)**			
Underweight	4.86663	0.40333	<0.0001
Overweight	−3.41565	0.18468	<0.0001
Obese	−4.72494	0.23981	<0.0001
**Smoking (Ref: Never)**			
Former	−0.16315	0.23373	0.4852
Current	−2.06691	0.23946	<0.0001
**Drinking (Ref: Never)**			
Former	−0.41375	0.43681	0.3435
Current	5.15666	0.28677	<0.0001
**LDL-C (Ref: <130)**			
≥130	1.06535	0.14540	<0.0001
**Triglyceride (Ref: <150)**			
≥150	−8.52168	0.18091	<0.0001
**SBP (Ref: <120)**			
120–139	−0.18963	0.18194	0.2973
≥140	0.32769	0.30047	0.2755
**DBP (Ref: <80)**			
80–89	0.04997	0.20428	0.8068
≥90	0.24884	0.33034	0.4513
